# Cytomegalovirus Gastritis Mimicking a Gastroesophageal Junction Malignancy in an AIDS Patient: A Case Report

**DOI:** 10.7759/cureus.77770

**Published:** 2025-01-21

**Authors:** Connor Lovingood, Nancy Jhanji, William K Oelsner, James E Pitcher, Laxmi Parsa

**Affiliations:** 1 Internal Medicine, University of Tennessee College of Medicine - Chattanooga, Chattanooga, USA; 2 Gastroenterology, University of Tennessee College of Medicine - Chattanooga, Chattanooga, USA; 3 Gastroenterology and Hepatology, University of Tennessee College of Medicine - Chattanooga, Chattanooga, USA

**Keywords:** cytomegalovirus (cmv), gastritis, gastrointestinal tract ulcer, gi malignancy, hiv aids

## Abstract

Cytomegalovirus (CMV) gastritis is a rare opportunistic infection that often affects immunosuppressed patients. It is a DNA virus belonging to the Herpes family most commonly spread through contaminated bodily fluids such as blood, urine, saliva, tears, breast milk, semen, and vaginal fluids. Here, we present a case of a newly diagnosed HIV-positive patient with acquired immunodeficiency syndrome (AIDS) found to have a gastroesophageal (GE) junction ulceration with endoscopic characteristics suggestive of malignancy due to CMV gastritis in the absence of underlying malignancy. Most immunocompetent patients experience mild or no symptomatology and thus often require no treatment. On the contrary, immunosuppressed patients may be greatly affected by CMV including death. Because of this, it is necessary to treat and potentially prophylax against CMV within this population depending on the severity of immunocompromise and overall clinical suspicion. The most common treatments include ganciclovir, valganciclovir, cidofovir, and foscarnet. This case highlights the rare location and endoscopic appearance of CMV gastritis. Since the endoscopic appearance of CMV is highly variable, immunohistochemistry of biopsied mucosa is the only reliable method to correctly diagnose. In addition, this case illustrates the importance of maintaining a broad differential including rare and treatable diseases. Lastly, it summarizes the observed association of CMV ulcers at the GE junction with underlying malignancy reported in the literature.

## Introduction

Cytomegalovirus is a DNA virus that belongs to the Herpesviridae family with a wide seroprevalence range depending on the source of 40-100% [1]. CMV rarely affects immunocompetent individuals but usually presents with symptoms common in mononucleosis such as fever, chills, fatigue, lymphadenopathy, splenomegaly, nausea/vomiting, and general malaise. It is usually self-resolving within a few weeks. Immunocompromised patients from a variety of medical illnesses or undergoing certain treatments including those with HIV/AIDS and CD4 counts less than 50 are more commonly affected as the virus undergoes reactivation. Clinical presentations are typically more severe for these patients and may include esophagitis, gastritis, gastric ulcer, gastroparesis, pancreatitis, ileitis, appendicitis, proctitis, and colonic obstruction. The virus is more likely to undergo reactivation in the setting of immunocompromise leading to a wide range of clinical manifestations including those aforementioned along with encephalitis, pneumonitis, retinitis, and fulminant hepatitis [2]. The most common CMV gastrointestinal manifestation is colitis [1]. However, CMV gastritis has also been recorded in the literature, although much more infrequent [1,3-7]. In this case report, a newly diagnosed HIV-positive patient with AIDS was found to have a malignant appearing gastroesophageal junction ulceration due to CMV gastritis in the absence of esophagitis or underlying malignancy. The location of the lesion in this patient is what makes this case exceptionally rare as an isolated ulceration caused by CMV at the gastroesophageal junction in the absence of esophagitis has only been documented a handful of times in the literature. The objective of this case report is to highlight a rare variation of CMV gastritis.

## Case presentation

A 60-year-old woman with a past medical history of recently diagnosed HIV with AIDS, pancytopenia, B12 and folate deficiency, irritable bowel syndrome, colon polyps, and hypothyroidism presented with acute encephalopathy, gait instability, and failure to thrive. She was admitted with hyponatremia, anemia, and malnutrition. The patient had been diagnosed with HIV one month prior; however, she had not started antiretroviral therapy or infection prophylaxis. CMV does not routinely require empiric prophylaxis in AIDS patients given its overall low incidence. Her outpatient labs confirmed a diagnosis of AIDS with a CD4 count of 34, placing her at risk of several opportunistic infections including CMV, and HIV RNA >700,000 two weeks prior (Table 1).

**Table 1:** Laboratory investigations on admission.

Lab	Patient Values	Reference Range
Sodium	129 mmol/L	136-145 mmol/L
Potassium	3.5 mmol/L	3.5-5.1 mmol/L
Calcium	7.9 mg/dL	8.9-10.8 mg/dL
Chloride	98 mmol/L	98-107 mmol/L
Bicarb	25 mmol/L	22-31 mmol/L
BUN	15 mg/dL	7-26 mg/dL
Creatinine	0.4 mg/dL	0.6-1.1 mg/dL
Glucose	79 mg/dL	80-99 mg/dL
AST	11 U/L	5-34 U/L
ALT	20 U/L	3-55 U/L
ALP	72 U/L	40-150 U/L
T. Protein	6.3 g/dL	6-8.3 g/dL
Albumin	2.7 g/dL	3.5-5 g/dL
T. Bilirubin	0.4 mg/dL	0.2-1.2 mg/dL
Hemoglobin	7.7 g/dL	12-16 g/dL
WBCs	5 Th/mm3	4.8-10.8 Th/mm3
Platelets	314 Th/mm3	130-400 Th/mm3
INR	1.01	1.00
APTT	29 Sec	25.1-36.5 Sec
Iron saturation	11.7%	20-50%
Serum Iron	19 ug/dL	40-145 ug/dL
TIBC	163 ug/dL	226-426 ug/dL
Transferrin	124 mg/dL	191-379 mg/dL
Ferritin	584.5 ng/dL	11-306.8 ng/dL
LDH	172 U/L	117-242 U/L
Haptoglobin	380 mg/dL	33-346 mg/dL
Ammonia	41 umol/L	16-53 umol/L
TSH	12.042 uIU/mL	0.34-5.60 uIU/mL
T4, free	0.57 ng/dL	0.54-1.24 ng/dL
CD4	34 cells/uL	359-1519 cells/uL
HIV-1 RNA PCR	788,000 copies RNA/mL	<20 copies RNA/mL
Cryptococcus	NEGATIVE	NEGATIVE
Hepatitis panel	NON-REACTIVE	NON-REACTIVE
RPR	NON-REACTIVE	NON-REACTIVE

**Figure 1:**
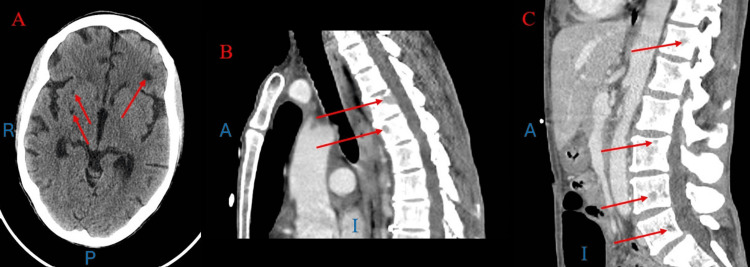
Panel A: Hypodensities in the L frontal and R basal ganglia regions from CT brain without contrast. Panels B and C: Numerous lytic lesions concerning for disseminated infection vs metastatic disease. Panel B at the levels of T4-T5 and Panel C at the levels of T12, L3-L5 from CT chest, abdomen, and pelvis with contrast. Blue letters represent the orientation of the image; R: Right; P: Posterior; A: Anterior; I: Inferior

At the time of admission, labs were notable for sodium of 129 and hemoglobin of 7.7 with a previous baseline of around 10 (Table 1). CT brain showed focal hypodensities in the left frontal region and right basal ganglia; however, MRI was negative for demyelination or other acute abnormalities (Figure 1A). CT chest/abdomen/pelvis showed numerous lytic lesions concerning metastatic disease, later discovered to most likely be related to disseminated infection with CMV as they were not present in the CT scan two months prior (Figures 1B, 1C). The patient reported difficulty eating due to nausea and dysphagia. A review of systems was negative for vomiting, fevers, diarrhea, constipation, melena, hematochezia, or hematemesis. The patient denied any NSAID, anticoagulation, or antiplatelet use. She reported no alcohol, smoking, or illicit drug use. Her last colonoscopy was three years prior with multiple small polyps, and she had no prior esophagogastroduodenoscopy (EGD). During admission, she was evaluated by infectious disease and started on fluconazole 200mg daily for seven days for oropharyngeal candidiasis as well as trimethoprim-sulfamethoxazole DS daily for P. jirovecii prophylaxis and a multivitamin.

**Figure 2:**
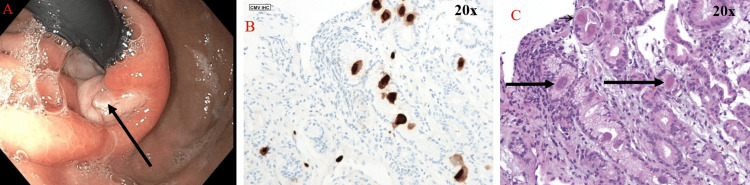
Panel A showing gross depiction via endoscopy of malignant-appearing, non-bleeding, cratered ulceration measuring 2mm in largest dimension at GE junction. Panel B 20x photomicrograph of biopsied ulcer with immunohistochemical staining for CMV inclusion bodies. Panel C 20x photomicrograph of biopsied ulcer with CMV inclusion bodies. GE: Gastroesophageal; CMV: Cytomegalovirus

Gastroenterology was consulted for further evaluation of failure to thrive and dysphagia. She endorsed a 55-pound weight loss over three months in addition to iron deficiency anemia and epigastric pain. An EGD was performed and showed a normal-appearing esophagus with erythematous mucosa throughout the stomach along with a large ulceration at the GE junction with findings concerning for malignancy including a rolled, irregular border, seemingly deep tissue invasion, and pale color (Figure 2A). Pathological examination revealed CMV inclusion bodies without evidence of malignancy (Figures 2B, 2C) and the patient was started on IV valganciclovir 900mg twice daily for six weeks and proton-pump inhibitor therapy daily. At this time, she also began HIV treatment with bictegravir 50mg/emtricitabine 200mg/tenofovir alafenamide 25mg.

The patient was discharged to a skilled nursing facility with a subsequent presentation two weeks later for recurrent falls and failure to thrive. The patient elected to transition to hospice care at that time and forwent additional work-up.

## Discussion

Immunocompromised patients with HIV have nearly double the lifetime risk of esophageal and gastric cancers as CMV proteins are often present in cancer cells and have been shown to cause epithelial transformation via the presence of oncogenes, increase local inflammation, and promote tumor angiogenesis [8]. CMV has also been shown to be present in the prostate, breast, B-cell lymphoma, and glioblastoma [9]. However, opportunistic infections can often present with signs and symptoms similar to malignancy in patients with severe immunosuppression [5]. Here, we present a case of a malignant appearing GE junction ulceration that was found to be CMV gastritis. Mucosal ulceration is typically caused by local invasion into the endothelium leading to vasculitis, ischemia, and tissue necrosis.

CMV gastritis is extremely rare and a largely undiagnosed disease, often found in the setting of disseminated infection [1]. In advanced disease, CMV is known to create pseudo-malignancies and ulcerations [10,11]. While CMV colitis is the predominant clinical phenotype, upper GI manifestations have also been reported [1,6]. One retrospective study by Marques et al. found the gastric antrum (42%) and lower esophagus (33%) to be the most common sites of CMV disease in the upper GI tract [3]. The location of our patient’s ulceration at the GE junction is exceedingly rare.

Currently, there are few case reports of CMV ulcerations at the GE junction. However, of the three reports in the literature, one patient had a CMV pseudo-malignant mass, while the other two patients had an underlying hematologic malignancy [12,13]. While our patient was not diagnosed with malignancy, she was subsequently found on re-hospitalization to have lytic bone lesions, suggestive of an opportunistic infection versus undiagnosed malignancy. While CMV is currently not known to have malignant transformation, it has been shown to be a risk factor for post-transplant lymphoproliferative disease in conjunction with Epstein-Barr virus infection [14]. It is interesting to note the observed association of CMV ulcers at the GE junction with underlying hematologic malignancies.

## Conclusions

In summary, this case report highlights the importance of maintaining a broad differential and considering CMV gastritis for gastric ulcerations in immunocompromised patients, particularly those with advanced HIV or T-cell dysfunction. The rarity of our patient’s lesion at the GE junction underscores the need for heightened clinical suspicion and thorough evaluation given its potential to mimic malignancy. Moreover, CMV reactivation is a known risk factor for lymphoproliferative disease, and patients with CMV gastritis may have concurrent hematologic malignancies necessitating repeat endoscopy and biopsy pending overall clinical suspicion as no current guidelines exist at this time. Lastly, this case emphasizes the importance of early initiation of antiretroviral therapy in HIV-positive patients to reduce the risk of opportunistic infections and improve overall outcomes.
